# Immune Response and COVID-19: A mirror image of Sepsis

**DOI:** 10.7150/ijbs.48400

**Published:** 2020-07-09

**Authors:** Eduardo López-Collazo, José Avendaño-Ortiz, Alejandro Martín-Quirós, Luis A. Aguirre

**Affiliations:** 1 The Innate Immune Response Group, IdiPAZ, La Paz University Hospital, Paseo de la Castellana 261, Madrid 28046, Spain.; 2 Tumor Immunology Laboratory, IdiPAZ, La Paz University Hospital, Paseo de la Castellana 261, Madrid 28046, Spain.; 3 CIBER of Respiratory Diseases (CIBERES), Madrid, Spain.; 4 Emergency Department and Emergent Pathology Research Group, IdiPAZ La Paz University Hospital, Paseo de la Castellana 261, Madrid 28046, Spain.

**Keywords:** COVID-19, Sepsis, Immune response, Immune-checkpoints, T-cell exhaustion

## Abstract

The emergence of SARS-CoV-2 virus and its associated disease COVID-19 have triggered significant threats to public health, in addition to political and social changes. An important number of studies have reported the onset of symptoms compatible with pneumonia accompanied by coagulopathy and lymphocytopenia during COVID-19. Increased cytokine levels, the emergence of acute phase reactants, platelet activation and immune checkpoint expression are some of the biomarkers postulated in this context. As previously observed in prolonged sepsis, T-cell exhaustion due to SARS-CoV-2 and even their reduction in numbers due to apoptosis hinder the response to the infection. In this review, we synthesized the immune changes observed during COVID-19, the role of immune molecules as severity markers for patient stratification and their associated therapeutic options.

## Introduction

The emergence of the new coronavirus, known as SARS-CoV-2, poses a substantial threat to public health and a major impact to worldwide economies and societies. The infection caused by this virus leads to a primary viral pneumonia identified as COVID-19, which resembles the Severe Acute Respiratory Syndrome (SARS) 1. Despite similarities with the seasonal influenza, severe disease behaves quite differently. The data indicate that 80% of COVID-19 infections are mild or asymptomatic, while approximately 15% are severe and require oxygen supplementation, and 5-10% are critical, characterized by SARS with acute respiratory distress syndrome (ARDS) and requiring mechanical ventilation in an intensive care unit 1-4. These subgroups of severe and critically infected patients are larger than those observed for influenza infection 5. Due to the rapid spread of COVID-19, affecting almost 200 countries, the World Health Organization (WHO) announced on March 11^th^ the elevation of COVID-19 from an epidemic to a pandemic, raising this infection to a global health priority 6, 7. Accordingly and to avoid the spread of the disease, several governments have implemented extraordinary measures, such as declaring states of emergency and instituting quarantines 8.

According to data obtained from numerous cohorts, the main causes of death by COVID-19 include respiratory failure and the onset of sepsis. In fact, sepsis has been observed in nearly all deceased patients in many of the reported cohorts 9-12. However, the sepsis figures were not always related to the bacterial findings in the microbiological work-up, suggesting SARS-CoV-2 as the etiological agent causative of this systemic condition 13. Additionally, comorbidities such as diabetes, hypertension and coronary disease and factors such as age, procalcitonin and interleukin (IL)-6 levels, leukocytosis and lymphocytopenia have been included as associated with mortality in patients with COVID-19 9, 14. Abnormalities in these factors in patients with unfavorable progression, combined with the high incidence of sepsis, strongly suggest the involvement of significant changes in the host's immune response. Additionally, evidence suggests the potential role of immune receptors such as Toll-like receptors (TLRs) and dipeptidyl peptidase 4 in the hijacking and virulence of the infection 15-17.

Current treatments employed against SARS-CoV-2 include a number of antiretroviral therapies recycled from other infections such as ribavirin, lopinavir/ritonavir, oseltamivir, hydroxychloroquine and remdesivir 18, 19, inflammation modulators and anticoagulants 20. Their usefulness, however, has led to major public debate. According to a randomized trial on hydroxychloroquine as post-exposure prophylaxis for COVID-19, the compound did not prevent the disease when administered within 4 days after exposure to the virus 21. Although remdesivir has shown significant activity both *in vitro* and in a primate model against SARS-CoV-2 22-24, the drug has only provided moderate clinical benefit for the treatment of patients with COVID-19 25, 26. Thus, the lack of a successful treatment and the absence of vaccines has prompted the scientific community to explore other avenues. Host-directed therapies such as immunomodulators could be an interesting alternative for treating patients with COVID-19.

In this review, we summarize the immune changes observed in SARS-CoV-2 infection, with an emphasis on the similarities with sepsis, and the role of immune compounds as severity markers and therapeutic targets.

## Coronavirus heterogeneity

SARS-CoV-2 is a β-coronavirus included in the sarbecovirus subgenus, orthocoronavirinae subfamily, which is broadly distributed in humans and other mammals 7. The first human coronaviruses (HCoV-229E and HCoV-OC43) were described in the 1960s, while HCoV-NL63 and HCoV-HKU1 (which cause mild infections in immunocompetent individuals) were described in the early 2000s 27. However, SARS-CoV1 (SARS1) and MERS-CoV (MERS), the other two members of the family that have affected humans in this century, are highly transmissible and pathogenic. In fact, the two pathogens have caused more than 700 deaths in 27 countries during their respective outbreaks in 2002 and 2012 28.

SARS-CoV-2 shows close similarities with SARS1 and MERS in terms of pathogenicity and has largely overtaken the death toll of the latter two. As with SARS1 and MERS 29, SARS-CoV-2 presents a serious risk of infection for healthcare workers on the frontlines 30, which is likely due to nosocomial transmission 31 given the substantial virus shedding that only occurs after the onset of symptoms (i.e., when patients are already hospitalized for days) and the fact that the viruses can remain on hospital surfaces for several days after patients no longer test positive 32-34.

Since they belong to the order *Nidovirales*, the viruses' molecular structures show similarities, although there are slight differences, which will be discussed below. Basically, SARS-CoV-2 is an enveloped, non-segmented, positive-sense RNA virus that has four main structural proteins: the spike (S) glycoprotein, the small envelope (E) glycoprotein, the membrane (M) glycoprotein, and the nucleocapsid (N) protein. In addition, SARS-CoV-2 has several accessory proteins that are relevant to the host immune process, as will be discussed below.

## SARS-CoV-2 entry points

According to reported data, angiotensin-converting enzyme 2 (ACE2) has been identified as a functional receptor for SARS1 35. ACE2 is highly expressed in several tissues including myocardial cells, kidney proximal tubule cells, bladder urothelial cells, testis cells and lung cells 36-38. In lung tissue, ACE2 is mainly located on the apical side of the epithelial cells in the alveolar space 39, 40. SARS1 primarily infects ciliated bronchial epithelial cells, unlike MERS, which primarily infects unciliated bronchial epithelial cells by binding to dipeptidyl peptidase 4 (CD26) receptors 27. This difference is due to each virus' differing structure for the receptor-binding domains (RBD) of S proteins. While the SARS1 RBD contains a receptor-binding motif (RBM) rich in loops, MERS-RBD has an RBM that contains a four stranded β-sheet 27.

Structural and functional analyses have shown that SARS-CoV-2 also binds to ACE2 41-43. Given that the SARS-CoV-2 protein S interacts with ACE2 expressed on type II pneumocytes in the lungs 44, ACE2 has been postulated as the entry point of this virus into pneumocytes. Dipeptidyl peptidase 4 15 and CD147 45 have also been reported as entry points for SARS-CoV-2.

Lung-resident macrophages are located on the apical side of the epithelium and, together with dendritic cells, act as innate immune cells to attack viruses until adaptive immunity is involved. It is still an open question as to how SARS-CoV-2 interacts with and eventually enters immune cells. Both ACE2 and dipeptidyl peptidase 4 are expressed in these cells but to a limited extent 46-50. However, phagocytosis of virus-infected apoptotic epithelial cells by resident macrophages and the presence of other proteins binding to SARS-CoV-2 could be pathways of interaction between the cells and SARS-CoV-2.

Successful virus-host cell fusion requires other molecules. Several research groups have attempted to inhibit host proteases, such as cathepsins and TMPRSS2, a protease that processes S protein and favors virus entry into cells 51. Other authors have demonstrated that camostat mesylate, an inhibitor of TMPRSS2 protease activity, partially inhibited the entry of SARS-CoV-2 into primary lung epithelial cells 44.

## The immune response in COVID-19

In terms of the circulatory system, several reports have demonstrated a direct association between proinflammatory cytokine levels in plasma and lung injury during infection by coronaviruses such as SARS1 52 and MERS 53. Changes in the inflammatory response have explained the high impact of MERS on infected patients with diabetes 9. SARS-CoV-2 has also been shown to severely affect cytokine levels 9, 14.

Innate immune system (IIS) cells, mainly monocytes and macrophages, are the principal players in orchestrating the host's inflammatory response by activating the nuclear factor kappa B (NF-κB) and interferon regulatory factor (IRF) pathways 54, 55. As previously reported for sepsis, patients with severe COVID-19 infections show excessive inflammation and cytokine storms, including overexpression of interleukin (IL)-1β, IL-2, IL-6, and tumor necrosis factor alpha, in the early phase of the disease (**Table 1**) 56. These hallmarks of sepsis have been widely explained by an exacerbation of macrophage activation 57.

According to several studies, the inflammatory phase for patients with severe COVID-19 is limited to the initial period of the disease 7. The subsequent chronic basal inflammation, which lasts several days, leads the immune system towards a refractory state, which is also observed in protracted sepsis. A comparative study of patients with severe and mild COVID-19 demonstrated that all cytokines, except IL-6 and IL-10, reached their peak serum levels 3-6 days after disease onset. IL-6 levels began to drop approximately 16 days later, and IL-10 levels were at their lowest 13 days after disease onset. Interestingly, the cytokine levels reached similar points for all patients with severe and mild disease 16 days after disease onset 56. This phenomenon mirrors the most advanced phases of sepsis, when macrophages develop a refractory state characterized by strong inhibition of the NF-κB and IRF pathways in response to pathogens 54, 55, 57. However, little is known about the role of the TLR family in this context. Authors have postulated that SARS1 infection regulates immune-related genes in myeloid cells by TLRs 58, which might be important to the pathogenesis of SARS. Other authors have demonstrated that mice deficient in the TLR3/TLR4 TIR-domain-containing adapter-inducing interferon-β (TRIF) were highly susceptible to SARS1 infection 59. Both SARS1 and SARS-CoV-2 require acidification of endosomes and lysosomes to infect cells 24, 60. A number of authors have suggested that the ssRNA of SARS-CoV-2 could bind TLR3/7/8 resulting in the induction of type I interferon (IFN-I) 61. Therefore, the overactivation of inflammatory signaling points to the important role of TLRs in SARS-CoV-2.

Various clinical trials have included IL-6 and IL-6R-blocking antibodies to prevent this anaphylactic toxicity observed in patients with COVID-19 5, 56. In this context, however, anticytokine-based therapies might control only the cytokine storm without deleterious effects on virus replication. In addition, colony stimulation factors (CSFs) show significantly aberrant overexpression in patients with COVID-19. High granulocyte-macrophage (GM)-CSF levels have been detected in circulating lymphocyte populations, excluding natural killers (NKs) and B cells, from patients with COVID-19 admitted to intensive care units 62. Similarly, a number of studies on sepsis have shown that GM-CSF deficiency protects mice in models of lethal endotoxemia 63. Thus, the potential effect of GM-CSF-blocking antibodies as a treatment for SARS-CoV-2 is being evaluated by research groups and pharmaceutical companies (NCT04351152 and NCT04341116) 1, 64, 65. Other current strategies undergoing clinical investigation for reducing macrophage activation include the blockade of certain cytokines, inhibition of CCR5-mediated migration and CD14 blockade by monoclonal antibodies (NCT04348448, NCT04324021, NCT04345445, NCT04347239 and NCT04346277) 66, 67.

An increasing number of reports have indicated that not only the IIS but also the adaptive system becomes deregulated during COVID-19 infection. The approximately 83% of patients who have shown lymphocytopenia at admission illustrates this point 10, 56. Although macrophages appear to play a major role in the early phase of the pathophysiology of SARS-CoV-2, the adaptive immune system likely emerges as a crucial factor in the late phase. These observations however apply only to those patients who require hospitalization and clinical care. Approximately 80% of individuals infected with SARS-CoV-2 are asymptomatic or experience mild symptoms 1-4.

A number of analyzed cohorts have confirmed that the severe phase appears in these patients approximately 8-11 days after the onset of the disease, when proinflammatory cytokines reach their peak expression, probably due to an exacerbated innate immune response 56. However, the total number of lymphocytes is significantly reduced in those patients with a poor prognosis 56. A multicenter retrospective study showed that the lymphocyte count was an independent high-risk factor associated with COVID-19 progression 68. Other researchers have found an inverse correlation between lymphocyte counts and time to symptom reappearance in a cohort of patients with COVID-19 discharged from hospital 69.

Similarly, an obvious depletion of lymphocytes during sepsis has been demonstrated, which compromises the adaptive response in the second phase of the disease 70. Several researchers have studied this phenomenon, indicating a strong implication of immune checkpoints (ICs) and their ligands, such as the programmed death-1 (PD-1) and PD-L1 axis 71. Furthermore, PD-1/PD-L1 interaction during sepsis induces not only apoptosis but also lymphocyte “exhaustion”, an effect that can be reversed by blocking monoclonal antibodies against either PD-1 or PD-L1 72, 73. SARS-CoV-2 has observed to induce apoptosis of peripheral blood lymphocytes via *P53* activation 74, and PD-1 75 has been found to be upregulated in the late phase of COVID-19 infection. Interestingly, PD-1 overexpression has also been reported in other infections by retroviruses such as the lymphocytic choriomeningitis virus and human immunodeficiency virus. Moreover, there are a number similarities between HIV and SARS-CoV-2 in terms of origin, aggressiveness to human hosts but not animal hosts, etc., which might help in finding new ways to combat SARS-CoV-2 76. Other ICs, such as PSGL-1, play an important role in the pathophysiology of chronic viral infections 77.

**Figure 1:**
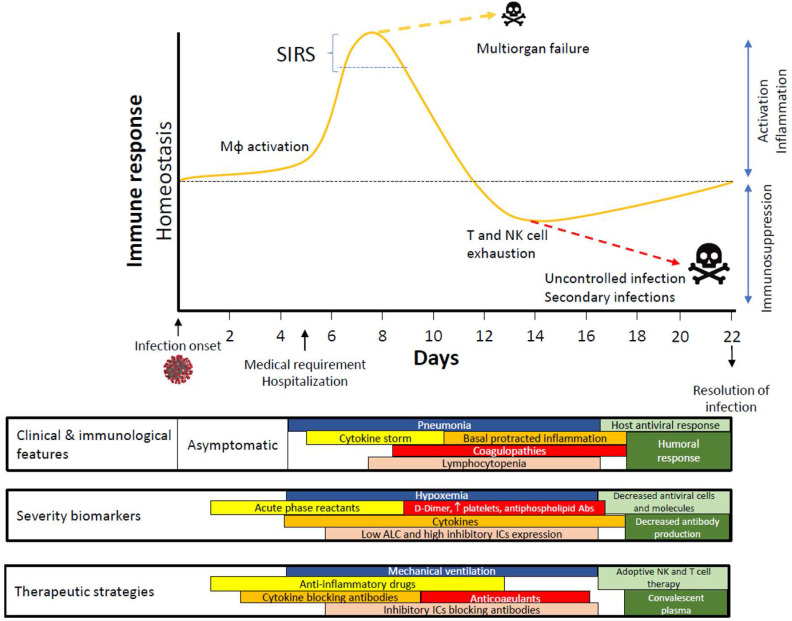
** Time course proposed for immune response in COVID-19.** Abbreviations: Abs, antibodies; ALC, absolute lymphocyte counts; Mφ, Macrophage/Monocyte; SIRS, systemic inflammatory response syndrome.

The collective data available point to an exaggerated innate response followed by an inappropriate switch to the adaptive response, which leads to immune system exhaustion during SARS-CoV-2 infection (**Fig. 1**). Likewise, the potential role of ICs and their ligands in lymphocytopenia observed in patients with severe COVID-19 should be a focus of research on SARS-CoV-2 infection. There is a crucial need for thorough studies on the development of clinical tools for personalized medicine for this infection. IC levels and IC-ligand expression would not only help physicians stratify patients at admission but also serve as pharmacological targets for those patients with a poor prognosis. Due to the impact of IC inhibitors in cancer therapies, several antibodies against ICs have been approved by the US Food and Drug Administration for use in certain malignancies 78, 79. However, only camrelizumab (a PD-1 monoclonal antibody) and thymosin have so far been included in a clinical trial (NCT04268537) for COVID-19 treatment 80, 81.

## Interferons and SARS-CoV-2

Previous data from animal models of SARS1 and MERS infection have shown a significant delay in IFN-I production by macrophages, which leads to lethal pneumonia in mice 82, 83. SARS1 and MERS encode at least 8 proteins that interact with the signaling cascades downstream of pattern recognition receptors, some of which suppress IFN signaling 28, 76. Several MERS proteins can inhibit NF-κB and IRF pathways, promoting an evasion of the innate immune response 84. Nevertheless, other authors have published conflicting results. While IFNβ-1b was shown to reduce disease severity and mean viral loads in necropsied lungs and extrapulmonary tissue in MERS 85, IFNα-2b neither decreased mortality nor accelerated viral clearance in a retrospective observational clinical study 86. IFNγ treatment, however, showed a protective role in lethal respiratory disease in mice infected by SARS1 87. Other authors have demonstrated that the beneficial or detrimental role of IFN-I depends on the timing of the SARS1 infection, indicating that IFN-I and inflammatory macrophages (IMMs) promote lethal SARS-CoV1 infection and identifying IFN-I and IMMs as potential therapeutic targets in patients infected with pathogenic coronavirus 82. Several combinations of antiretrovirals and human recombinant IFNs (such as IFNβ-1b and IFNα-2b) are currently employed for managing COVID-19 88, 89. Further research on IFNs for managing COVID-19 is therefore warranted.

The IFN-I response also has a key role in NK-cell activation and, subsequently, on triggering their antitumoral abilities. NK cell levels have been found to be reduced and to present an anergic status in patients with COVID-19 90. Chimeric antigen receptor-engineered NK cells are also being tested for treating COVID-19 (NCT04344548 and NCT04324996) 91, 92.

## Antibody and plasma therapy

Similar to common acute viral infections, the antibody profile against SARS1 has a typical pattern of immunoglobulin M (IgM) and IgG production. The kinetics reported for SARS1-specific IgM antibodies indicate that IgMs disappear after 12 weeks of disease onset. However, IgGs can last a long time and play a protective role 93. Little is known about this scenario for SARS-CoV-2. There have been several convalescent patients who donated plasma against SARS-CoV-2, as was the case for both SARS1 94 and MERS 95 at the start of their respective outbreaks. A study conducted with 10 adult patients with severe COVID-19 showed that one 200-mL dose of plasma was well tolerated by the patients, leading to the disappearance of viremia in 7 days, while the clinical symptoms and paraclinical criteria rapidly improved within 3 days 96. Cross-reactivity between antibodies generated against SARS1 and SARS-CoV-2 has also been reported 42, 44, 97. Although this cross-reactivity might jeopardize the detection by serological tests of patients with COVID-19, it could favor the resolution of the infection in certain individuals.

Generating recombinant human monoclonal antibodies could be another method for neutralizing SARS-CoV-2. CR3022, a SARS coronavirus-specific human monoclonal antibody, has been reported capable of binding to SARS-CoV-2-RBD 98. Monoclonal antibodies that neutralize SARS1, such as CR3014, could be a treatment option for SARS-CoV-2 infection 99. S309, an antibody previously identified in a patient who contracted SARS1 in 2003, appears to be promising candidate for neutralizing SARS-CoV-2 100.

## Coagulation abnormalities: another effect of dysregulation of the innate immune response with a mirror image in sepsis

Since the 1993 report on tissue factor (TF) synthesis by activated macrophages 101, several studies have demonstrated the association between inflammation and coagulation 102. During inflammation, macrophages contribute to disseminated intravascular coagulation (DIC) in numerous clinical contexts. Aberrant *in vivo* TF expression plays a pivotal role in the activation of blood coagulation in the setting of sepsis and endotoxemia 103, and the high incidence of sepsis-associated DIC is well known. TF expression provokes widespread thrombosis in the microcirculation of various organs, contributing to multiple organ dysfunction, a major determinant of mortality in sepsis 104-106. Peripheral blood mononuclear cells have shown obvious overexpression of coagulation-related genes in COVID-19 infection. In fact, a DIC score ≥5 points has been associated with mortality in patients with COVID-19, and more than 70% patients who die from the infection meet the International Society of Thrombosis and Hemostasis criteria for a DIC 107. Furthermore, anticoagulant treatment has been associated with hospital survival in hospitalized patients with COVID-19 108.

Proinflammatory cytokines induce the production of C-reactive protein (CRP) and haptoglobin by hepatocytes. A number of reports have indicated high CRP and haptoglobin levels in patients with COVID-19, with higher levels in those patients with a severe condition 10, 109-111. The presence of these acute-phase proteins has been associated with D-dimer levels, a fibrin cleavage product that serves as a biomarker of pulmonary embolism 112. A study indicated that patients with COVID-19 who died showed higher D-dimer levels and lower platelet counts than those who survived 9. Longer prothrombin and activated partial thromboplastin times have also been associated with poorer outcomes in COVID-19 infection 107. To prevent these abnormalities, anticoagulant heparin, tinzaparin and enoxaparin are being tested in patients with COVID-19 (NCT04344756 and NCT04345848).

## Outlook

COVID-19 has emerged as a complex disease that shares clinical characteristics with sepsis. **Figure 1** summarizes the progression of infected patients who usually require hospitalization from days 5 to 6 since the start of their infection. Depending on their immune response, patients might experience an exacerbated cytokine production that compromises their vital functions and leads to a state of exhaustion that hinders the activation of the adaptive response.

A number of published studies have reported the onset of symptoms compatible with pneumonia accompanied by coagulopathy and lymphocytopenia during COVID-19 infection. However, there are still no solid markers to predict these patients' progression. Cytokine elevation rates, the presence of acute phase reactants such as D-dimer, platelet activation and IC expression are some of the biomarkers proposed in this context. Given the similarity of COVID-19 infection to sepsis, it is possible that testing for the early expression of ICs and their ligands on innate immune cells such as monocytes and macrophages could serve as a tool for classifying patients on admission, thereby opening up new avenues for treatment. As has been observed in prolonged sepsis, T-cell exhaustion due to SARS-CoV-2 and even reduced T-cell counts due to apoptosis hinder the host's response to infection. In such a scenario, new infections might emerge, increasing the risk of mortality.

**Figure 2:**
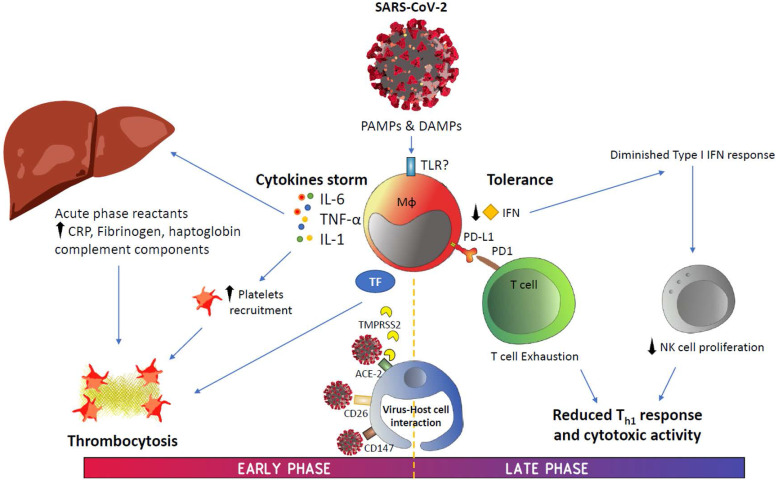
** Graphical summary.** Abbreviations: ACE-2, angiotensin-converting enzyme 2; CRP, C-reactive protein; DAMPS, danger-associated molecular patterns; IFN, interferon; IL, interleukin; PAMPS, pathogen-associated molecular patterns; PD-1, programmed cell death protein-1; PD-L1, programmed death ligand-1; TF, tissue factor; TMPRSS2, transmembrane serine protease 2; TNF-α, tumor necrosis factor alpha.

According to data generated on COVID-19 since its emergence, this disease has shown two potentially overlapping phases (**Figure 2**). The first phase is strongly characterized by a disproportionate IIS reaction that causes a cytokine storm and, subsequently, generates significant damage to the body. In addition, inflammation induces the production of a significant number of factors related to the coagulation cascade, resulting in the onset of thrombi and associated DIC, a condition that highly resembles that observed during sepsis.

The urgency and severity of COVID-related events in various countries is facilitating a significant number of clinical trials (**Table 2**). To date, we have only partial results, and exact answers have not been found in the clinics. In fact, there have been cases of conflicting results from clinical trials and on the use of drugs that have had deleterious effects 86, 113-117.

An in-depth study of the interaction between the virus and the immune system is warranted to identify appropriate therapeutic targets. We need to achieve a finely tuned balance between regulating the first wave of cytokines and reactivating an appropriate adaptive response, a delicate balance struck between simultaneously blocking and unblocking the immune response. This balancing act will require biomarkers that clearly show the proper approach. It is likely that IC ligands (already known or to be discovered) are overexpressed from the onset of COVID-19 infection. As mentioned previously, IC ligand levels could help stratify patients during admission and prevent the disease from progressing. A massive international study approach could be useful for developing this tool. Given that COVID-19 is a dynamic disease, however, we will need to study patients at different stages of the disease to identify predictive factors and appropriate targets. A single picture of cytokine levels, cell population distribution, and other markers of interest would only give us a partial idea of an evolving disease.

## Conclusion

Based on the evidence presented in this article, the study of the immune system and the coagulation cascade during COVID-19 infection could provide valuable information for approaching the diagnosis and treatment of this disease. There are many questions that remain unanswered in the context of COVID-19, including “Can we predict the progression of patients with COVID-19 by establishing their immune profiles on admission?”, “Could IC expression regulate the second phase of COVID-19?” and “Can IC inhibitors and their ligands be useful for COVID-19 therapy as they have been in many types of cancers and as they have been postulated in sepsis?” Identifying those patients with poorer prognoses will therefore facilitate the development of accurate host-directed therapies.

## Figures and Tables

**Table 1:** Pathological hallmarks of COVID-19 and associated biomarkers

Immune System	COVID-19 Hallmark	Phenotype associated to severe patients
*Innate*	Cytokine storm and over inflammation	Neutrophilia
		↑ Acute phase reactants: Reactive C protein, fibrinogen, procalcitonin and haptoglobin
		↑ Basal IL-6
	Reduced antiviral cytotoxicity	↓ NKs Frequency
	Coagulopathy	↑ D-Dimer
		↑ Platelets
*Adaptative*	T cell exhaustion and reduced humoral response	Lymphocytopenia
		↓ T Lymphocyte counts
		↓ B Lymphocyte counts

**Table 2:** Proposed host-directed therapies against the most relevant pathological hallmarks of COVID-19

Pathological phenomenon	Treatment	Mechanism of action	Clinical trial	Toxicity and pharmacokinetic studies in humans
Cytokine storm and Mφ	Corticosteroids	Anti-inflammatory steroid hormones	Dexamethasone: NCT04327401 and NCT04325061. Methylprednisolone: NCT04329650 and NCT04343729. Prednisone: NCT04344288.	Yes. Approved for several pathologies
	Sirolimus	mTOR inhibitor, immunosuppressant	NCT04341675	Yes. Approved for preventing of transplant rejection and lyphangioleiomyomatosis
	MSCs infusion	BM, DP and UC and NestCell® MSCs for increasing anti-inflammatory environment	NCT04346368, NCT0402519, NCT04339660 and NCT04315987	No. Biological treatment. Phase I and II trials are currently recruiting patients
	Pyridostigmine	Acetylcholinesterase inhibitor. Enhances anti-inflammatory activity of α7-nAChR	NCT04343963	Yes. Approved for myasthenia gravis
	Canakinumab	IL-1β blocking antibody	NCT04348448	Yes. Approved for cryopyrin-associated periodic syndromes
	Anakinra	IR-1R blocking antibody	NCT04324021	Yes. Approved for rheumatoid arthritis
	Tocilizumab, Sarilumab	IL-6 blocking antibodies	NCT04345445 and ChiCTR2000030894	Yes. Approved for rheumatoid arthritis
	Emapalumab	IFN-γ blocking antibody	NCT04324021	Yes. Approved for haemophagocytic lymphohistiocytosis
	Eculizumab, IFX-1	C5 blocking antibodies	NCT04288713 and NCT04341116	Yes. Eculizumab approved for paroxysmal nocturnal hemoglobinuria. IFX-1 texted for hijdradenitis suppurativa
	Lenzilumab, TJ003234	GM-CSF blocking antibody	NCT04351152 and NCT04341116	Yes. Phase I trial in healthy volunteers
	IC14	CD14 blocking antibody	NCT04346277	Yes. Tested for sepsis
Immune tolerance and exhaustion	Ruxolitinib, Baricitinib	JAK inhibitors	NCT04348071, NCT04337359, NCT04338958 and NCT04320277	Yes. Ruxolitinib approved for myelofibrosis, polycythaemia vera and graft-versus-host disease. Baricitinib approved for rheuamatoid arthritis
	Leronlimab	CCR5 blocking antibody	NCT04347239	Yes. Phase I trial in HIV
	Camrelizumab	PD-1 blocking antibody	ChiCTR2000029806 and NCT04268537	Yes. Tested for relapsed/refractory classic Hodgkin lymphoma
	Thymosin α1	Recombinant Human Thymosin α1 (rhTh)	ChiCTR2000029806 and NCT04268537	Yes. Tested for hepatitis B and C and various types of cancer
	Expanded NK cell infusion	Allogenic and modified CAR NK cells	NCT04344548 and NCT04324996	No. Biological treatment. Phase I and II trials are currently recruiting patients
	APCs infusion	Lentiviral transfected DCs and APCs with 2019-nCoV for improving antigen presentation	NCT04276896 and NCT04299724	No. Biological treatment. Phase I and II trials are currently recruiting patients
	IFNα-2b	Immunomodulator	ChiCTR2000029308	Yes. Approved for hepatitis and hematological tumors
	IFNβ-1b	Immunomodulator	MIRACLE Study: NCT 02845843	Yes. Approved for multiple sclerosis
	IFN-γ	Immunomodulator	MIRACLE Study: NCT02845843	Yes. Approved for infections associated with chronic granulomatous disease and malignant osteopetrosis
	Convalescent plasma	Hyperimmune antibodies plasma	NCT04343755, NCT04347681 and NCT04345523	No. Biological treatment. Phase I and II trials are currently recruiting patients
Coagulopathy	Enoxaparin	Anticoagulant. Heparin derivative	NCT04345848	Yes. Approved for deep vein thrombosis and pulmonary embolism
	Tinzaparin	Anticoagulant. Low molecular weight heparin	NCT04344756	Yes. Approved for deep vein thrombosis
	Heparin	Unfractionated heparin	NCT04344756	Yes. Approved for several diseases
Host viral entry	Camostat	TMPRSS-2 inhibitor	NCT04321096	No. Phase I and II trials are currently recruiting patients
	Linagliptin	DPP4 inhibitor	NCT04341935	Yes. Approved for type 2 diabetes mellitus
	APNO1	Recombinant Human Angiotensin 2 Converting Enzyme Analogue (rhACE2)	NCT04287686	Yes. Phase I safety trial in healthy volunteers

Abbreviations: α7-nAChR, alpha-7 nicotinic receptor; APCs, antigen-presenting cells; BM, bone marrow; CAR, chimeric antigen receptor; CCR-5, C-C chemokine receptor type 5; DCs, dendritic cells; DP, dental pulp; GM-CSF, granulocyte macrophage colony-stimulating factor; IFN, interferon; IL, interleukin; MSCs, mesenchymal stem cells; mTOR, mammalian target of rapamycin; PD-1, programmed cell death protein-1; TMPRSS2, transmembrane serine protease 2; UC, umbilical cord.
